# Improved long‐term patient‐reported health and well‐being outcomes of early‐stage breast cancer treated with partial breast proton therapy

**DOI:** 10.1002/cam4.1881

**Published:** 2018-11-19

**Authors:** Sandra L. Teichman, Sharon Do, Sharon Lum, Theodore S. Teichman, William Preston, Shelly E. Cochran, Carlos A. Garberoglio, Roger Grove, Carol A. Davis, Jerry D. Slater, David A. Bush

**Affiliations:** ^1^ Department of Radiation Medicine Loma Linda University Medical Center Loma Linda California; ^2^ Department of Surgical Oncology Loma Linda University Medical Center Loma Linda California

**Keywords:** breast cancer, long‐term survivors, patient‐reported outcomes, proton radiation, quality of life

## Abstract

**Background:**

Because early‐stage breast cancer can be treated successfully by a variety of breast‐conservation approaches, long‐term quality of life (QoL) is an important consideration in assessing treatment outcomes for these patients. This study compares patient‐reported QoL outcomes among women with stage 0‐2 disease treated via lumpectomy followed by whole breast irradiation (WBI) or partial breast proton irradiation (PBPT).

**Methods:**

In this cross‐sectional study, 129 participants evaluated QoL several years post‐treatment by responding to subjective instruments, including established scalar questionnaires and self‐report measures. Responses were averaged between the two groups.

**Results:**

At 6.5 years (median) postdiagnosis, participants’ demographic, and clinical characteristics were similar. Patient‐reported outcomes were reported as mean scale scores for the two groups, all displaying significant differences favoring PBPT, including: cosmetic breast cancer treatment outcome scale (BCTOS) (PBPT mean 1.45, WBI mean 1.88, *P* < 0.001); breast pain (PBPT mean 1.30, WBI mean 1.67, *P* < 0.05); breast texture (BPT mean 1.44, WBI mean 1.91, *P* < 0.001); clothing fit (PBPT mean 1.06, WBI 1.46, *P* < 0.001); fatigue (PBPT mean 2.24, WBI mean 3.77, *P* < 0.002); impact of daily life fatigue on personal relations (OBPT mean 0.83, WBI mean 2.15, *P* < 0.001); and self‐consciousness (appearance dissatisfaction) (PBPT mean 1.38, WBI mean 1.77, *P* < 0.004).

**Conclusion:**

Patients’ responses suggest that PBPT is associated with improved overall QoL compared to standard whole breast treatment. These self‐perceptions are reported by patients who are 5‐10 years post‐treatment, and that PBPT may enhance QoL in a multitude of interrelated ways.

## 1 INTRODUCTION

In the United States, breast cancer affects 1 in 8 women.^1^ It threatens life and affects how women perceive themselves in appearance, desirability, and disability. Fortunately, owing to early detection practices, many cases present in early stages.

Most patients with early‐stage breast cancer (EBC) receive breast‐conservation therapy (BCT), usually lumpectomy followed by whole breast irradiation (WBI). Traditionally, WBI has been given via X rays administered over five to seven weeks, but in recent years accelerated schedules have emerged, with the total radiation dose delivered in as little as three weeks. Long‐term follow‐up studies (most dealing with five‐to‐seven‐week schedules) reveal excellent control and survival rates.^2^

**Figure 1:**
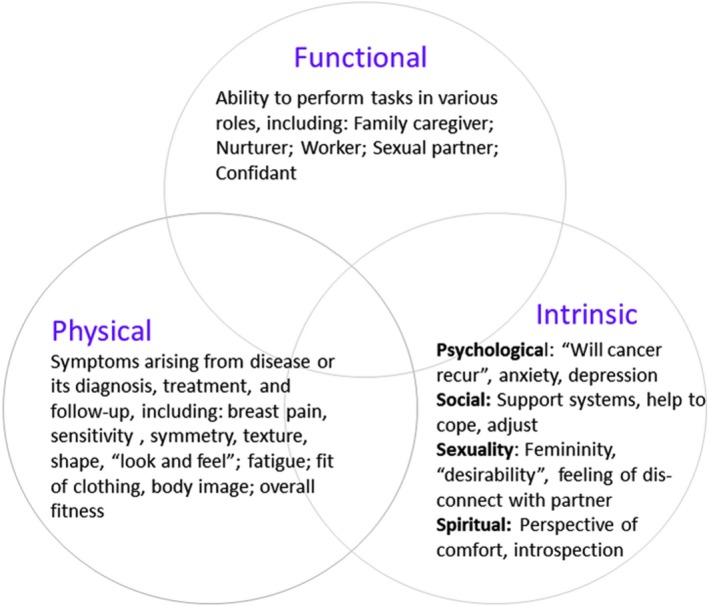
Schematic representation of interrelated factors influencing perceived QoL

Of 7.6 million female cancer survivors (as of 2014), more than 40% had breast cancer.^3^ Today, EBC patients may consider potential QoL in choosing therapy.^4^ Factors patients may consider in determining long‐term QoL include breast symptoms (sensitivity and pain), cosmesis, physical functioning (fatigue), emotional and social functioning, body image, and future perspective.^5^, ^6^ Breast cancer diagnosis and treatment can affect QoL in many ways. Figure 1 displays a model of how physical, functional, and intrinsic (ie, psychosocial) factors may interact to impact QoL.

The disease, its treatment, and resulting physical changes have led to radiotherapy approaches other than WBI, all aimed at reducing the extent and duration of normal‐tissue exposure. One approach is hypofractionated partial breast proton therapy (PBPT), offered at our institution^7^ and elsewhere.^8^ Other centers employ techniques delivered with X‐rays^9^, ^10^; in one of these, a phase III study^13^ of partial breast irradiation, preliminary results demonstrated equivalent outcomes in disease control and side effects, compared to WBI.^14^ Shorter treatment courses afford an option for patients deterred by the five‐to‐seven‐week course traditionally required for WBI.^15^

The purpose of our study was to determine whether long‐term differences in QoL obtained, given our experience with delivering WBI and PBPT for EBC. Since many acute radiation effects approach baseline within two years,^16^ we assessed patients approximately four to nearly 10 years post‐treatment to investigate whether significant differences in physical features and functioning prevailed long‐term. We hypothesized that such differences would influence patients’ daily activities, views of themselves, and relationships with others.

## 2 METHODS AND MATERIALS

### 2.1 Patient population

We performed a*post hoc* cross‐sectional survey study. Participants had EBC treated at LLUMC from 2003 to 2012 and received BCT, including PBPT or WBI. PBPT was administered via Phase II protocols initiated in 2003, which used protons for postlumpectomy irradiation to the tumor bed. Patients received 40 CGE (cobalt‐gray equivalent) in 10 daily fractions. Details of the study, from which most PBPT participants were drawn, are reported elsewhere.^7^, ^17^, ^18^ WBI consisted of 50 Gy X rays delivered to the entire breast, followed by a 10‐Gy boost to the tumor bed, delivered five days per week for approximately six weeks. This was standard‐of‐care treatment at our institution at the time.

Patients consented to researchers’ access to electronic medical records. Those were consulted to identify patients meeting eligibility criteria: a confirmed first‐diagnosis of EBC (stage 0‐2) receiving BCT, including PBPT or WBI; disease‐free survivors >5 years postdiagnosis; >age 40 at diagnosis; no chemotherapy (hormonal therapy permitted); tumor size ≤3 cm. Information also was abstracted from patients’ charts (disease stage, tumor characteristics, etc).

Participants received IRB‐approved packets comprising a cover letter describing the aims, voluntary nature of the study, requirements for participation (noting that a returned, completed questionnaire would be considered informed consent), and indicating that reminder letters and/or telephone calls might occur to answer questions; a current medical history/demographic form; questionnaires; a free‐response page; and a prepaid, preaddressed return envelope. Nine surveys were returned unopened. Follow‐up letters and calls were made as necessary.

### 2.2 Validated self‐administered tools

Given the overlapping nature of QoL domains (Figure 1), we selected general‐, modality‐, disease‐, and site‐specific instruments and self‐report measures intended to encompass the range of domains: Cosmesis (Harvard scale),^19^ a single‐item question asks one to rate the cosmetic result as excellent, good, fair, or poor^13^; Breast Cancer Treatment Outcome Scale (BCTOS),^5^ a treatment‐specific (BCT including radiotherapy), 22‐item questionnaire for subjectively evaluating functional and cosmetic outcomes^19^; Brief Fatigue Inventory (BFI), a nine‐item tool evaluating a common long‐term effect of treatment^20^ and severity of fatigue on a 0‐10 scale; Medical Outcomes Study (MOS) Short Form 20‐item Health Survey,^21^ assessing physical and mental health‐related QoL; Body Image Scale (BIS), developed with the European Organization for Research and Treatment of Cancer (EORTC),^22^ asking how respondents feel about their appearance and changes, which may have resulted from the disease or treatment, during the week prior to responding. Time required to complete these five tools was estimated to be 15‐20 minutes. For analysis, we separated the BCTOS into four subdomains as described by Stanton^5^: Breast Specific Pain, Functionality, Cosmesis, and Edema; the first three have been previously validated. In an attempt to validate whether results on this tool reflect factors patients consider most important, we asked them to indicate the top three questions/factors. This was a check to assure that differences observed through QoL studies such as ours really impact patients’ lives and directly affect patients’ individual experiences.

Additionally, participants were asked to indicate what they considered the three most significant questions on the Brief Fatigue Inventory, which were used as the basis of an individually tailored “weighted” subscale to confirm patient relevance. For each of the tools, the total score was taken as the sum total of all individual questions scores; this was used for all further analyses. For the subscales, the sum total of the subscore questions was used for analyses.

An investigator‐designed instrument featured nine general‐perspective questions that radiation oncology staff at Loma Linda University felt were relevant. Time required to complete this questionnaire was estimated to be five minutes. Further, an optional, open‐ended question, “What did we miss?” gave respondents opportunity to add whatever information they wished; we hoped to glean from the respondents some indications of factors they deemed important to QoL, but which our validated instruments might have failed to cover. We performed a brief analysis (reported herein) of responses to the first instrument; analysis for the former and latter will be reported separately.

### 2.3 Statistical methods

Analyses were performed using SPSS statistical software Version 22.0: (IBM Corp. Released 2013. IBM SPSS Statistics for Windows, Version 22.0. Armonk, NY: IBM Corp.) All reported *P* values were 2‐sided with *α* = 0.05. For categorical variables, we used Pearson correlations or Fisher's exact test, Student's *t* test. Student's *t* test, with equal variances assumed based on Levene's Test for Equality of Variances was used for individual questions, subscales, and total scores on the BCTOS, Brief Fatigue Inventory, Body Image Scale, Harvard Cosmesis Scale, and MOS Short Form. Additionally, the Student's *t* test with equal variances assumed was performed to compare demographic items between groups. A Pearson Chi‐Square was used to determine equivalent distributions between populations with regards to all demographic items. The Fischer's exact test and the Pearson Chi‐Square were used for analysis of question one on the Brief Fatigue Inventory.

Pearson correlations and covariance analyses were performed between age, months out from treatment, cosmesis score, all BCTOS subdomains (including our weighted subdomain and Stanton's experimental Edema subdomain), the Total Brief Fatigue Inventory Score, our Weighted Brief Fatigue Inventory Score, and the Body Image Scale total score. For correlation analyses of the Harvard Cosmesis Scale, the scale was inverted to align valence with the other scales, since the Harvard Cosmesis Scale defines a score of “1” to reflect poor cosmesis, while all other scales used in this study use the score of 1 to indicate the least adverse effects.

### 2.4 Administration

One hundred eighty patients who met eligibility requirements received surveys. Of these, 142 were completed (mostly at home; two at clinic; seven by telephone), yielding a 79% participation rate. Thirteen were ineligible owing to: bilateral disease^4^; disease recurrence^4^; recently diagnosed Stage 4 disease^2^; and serious medical comorbidities.^3^

## 3 RESULTS

Of the patients responding to the surveys, no significant differences were seen with regard to age, weight, marital status, race, adjuvant antiestrogen endocrine therapy, tumor size, nodal surgery, re‐excision, recent health status, employment history, education, or family history of breast cancer.

### 3.1 Baseline characteristics

The study comprised 129 responding women with EBC. The groups appeared to be matched across most domains (Table 1). Two exceptions were as follows: the ratio of Caucasian to non‐Caucasian patients was higher in the PBPT group (*P = *0.015); and “Time since diagnosis” was less in the WBI group. In the latter the median values for PBPT and WBI were 7, 6 (in years), respectively; the mean values were 7.3 and 6.2, respectively (in years), with standard deviations of 2.17 and 7.24, respectively (*P* < 0.989). The median age for the PBPT group was 72.5 years, and the median age for the WBI group was 70 years (not significant); the mean age was 64.7 years for the PBPT group and 62.9 years for the WBI group, with a standard deviation of 9.9 and 9.0, respectively (*P* < 0.307).

**Table 1:** Patient‐reported sociodemographic and clinical characteristics

Characteristic	PBPT (n = 72)	WBI (n = 57)	Significance
Age at survey			0.317^a^
Median, y (range)	72.5 (53‐94)	70 (46‐86)	
Mean, y	65	63.32	
Time since diagnosis (y)			
Median^b^	7	6	
Mean^c^	7.44	6.23	0.006^**,^ b ^,^ c
Race/Ethnicity			0.413^a^
Caucasian	60	35	
African American	3	2	
Hispanic	6	10	
Asian	3	9	
Native American		1	
Employed prior diagnosis			0.354^a^
Yes	37	30	
No	34	25	
Employed Currently			0.628^a^
Yes	20	16	
No	13	14	
Retired	39	27	
Fulltime (FT); Parttime (PT) Employment			0.489^a^
FT	13	14	
PT	6	2	
Education			0.529^a^
High School	15	17	
College	37	26	
Postgraduate	19	12	
Other	1	1	
Marital status			0.155^a^
Married	39	35	
Single/Divorced	5/12	7/3	
Widowed	16	12	
Stage^d^			
0	15	12	
I	48	38	
II	9	7	
Tumor size, cm			
Median (range)	1.37 (<0.01‐3.0)	1.24 (0.02‐2.8)	
Lymph node surgery^e^ (n/%)			
SLND^f^	68	55	
Level II (midaxilla)	2	0	
Additional surgery			
Re‐excision	18	14	
Wider margins, initial treatment	3	3	
Oncoplasty/Mammoplasty	3	0	
Involved breast			0.477^g^
Left breast	41	29	
Right breast	31	28	
Endocrine therapy^h^			
Currently taking	3	6	
Past	37	34	
Radiation duration impact^i^			0.001^a^
No	52	20	
Yes	19	37	
Out of town^j^			0.001^a^
Yes	32	8	
No	40	48	

a Pearson Chi‐square significance (2‐tailed).

b Student's *t* test significance (2‐tailed).

c Median values included as distribution can vary (Mean value affected by outliers).

d AJCC/UICC TNM Classification and Stage Groupings: 0 = TisN0MO; I = TINOMO; T1CN0M0; T2N0M0; T1bN0M0; T2N0M0; II = T2N0MO.

e Seven patients had microscopic (n = 4) or pathologic N1 (n = 3) disease.

f Sentinel Lymph Node Dissection (SLND); (2 PBPT & 2 WBI = no LN notation of biopsy).

g Fisher's Exact Test significance (2‐tailed).

h Question 15, Demographic form: Have you ever received hormone (antiestrogen) therapy? Yes/No; Are you currently taking hormone therapy? Yes/No _________ (blank provided for listing agent). Five answers indicated confusion: one woman left question blank; four women answered according to HRT (Premarin) or birth control prior use, not addressing antiestrogen question intent. Basic count only included for informational purposes.

i Convenience of care (impact on work, home duties); daily treatment duration (approximately 40 min); distance to radiation center; tx = treatments. PBPT: radiation therapy 5‐days per week, M‐F, delivered over 2 wk (10 treatments); WBI: radiation M‐F delivered over 6 wk (30 treatments) with boost to tumor area (in all but one patient).

j Defined as greater than 1 h away.

### 3.2 Patient‐reported cosmetic result in treated breast

**Figure 2:**
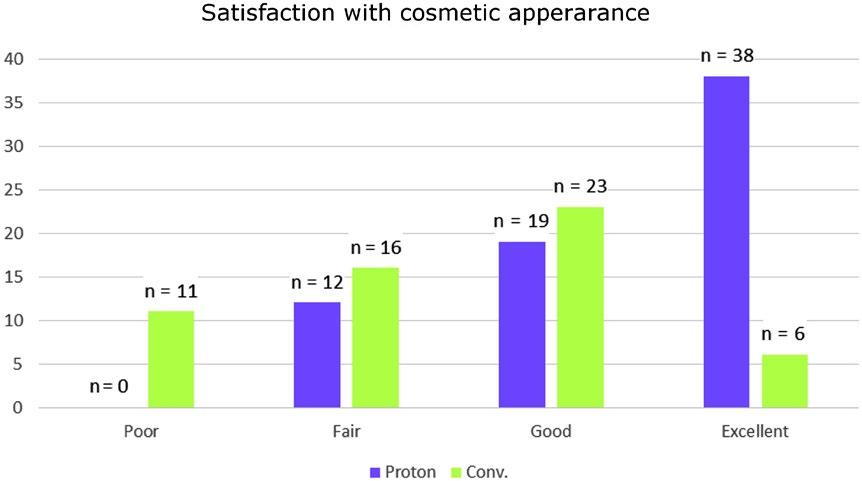
Frequencies of responses for cosmetic satisfaction as reported by patients, separated by treatment group. Patients were asked to respond according to the following scale: 1 = Poor; 2 = Fair; 3 = Good; 4 = Excellent. One patient receiving WBI and three receiving PBPT left this question blank

Patient‐reported cosmesis was more favorable in patients treated with PBPT (Figure 2). Overall mean values were found to be 3.40 and 2.44 for PBPT and WBI, respectively, and a standard deviation of 0.75 and 0.96, respectively, on a scale where 4 = “Excellent” and 1 = “Poor” (*P* < 0.001). In the PBPT group, one patient marked “2.5” (between 2 and 3 on the survey scale) and one patient marked “3.5” (sim.); these were rounded down to 2 and 3, respectively.

### 3.3 Patient‐reported BCTOS

Scores for each subdomain and the weighted domain are reported in Table 2. For the Cosmetic subdomain, the PBPT group mean score was 1.45 and the WBI mean was 1.88 (*P* < 0.001). On the Breast Specific Pain subdomain, the PBPT group mean score was 1.42 and the WBI mean was 1.25 (*P* < 0.005). On the Functionality subdomain, the PBPT mean was 1.11; the WBI mean was 1.17 (*P* < 0.311). For Edema, the PBPT mean score was 1.07; the WBI mean was 1.12 (*P* < 0.526).

**Table 2:** BCTOS. Tool Scores for four subdomains: comparison between treatment groups

BCTOS subdomains	Average domain scores (mean)		*P*‐value
	PBPT	WBI	
Weighted BCTOS^a^	1.84	2.55	<0.001***
Cosmetic BCTOS^b^	1.45	1.88	<0.001***
BS Pain BCTOS^c^	1.42	1.25	0.005**
Edema BCTOS^d^	1.07	1.12	0.526
Functionality^e^	1.11	1.17	0.311

Scale: 1 = None; 2 = Slight; 3 = Moderate; 4 = Large (Major).

a Generated by asking patients to circle three questions they thought most important. Scores were averaged for respondents and compared between treatment groups.

b Mean computed from items 1, 2, 4, 12, 13, 14, 20, 22

c Mean computed from items 7, 10, 21

d Mean computed from items 3, 9, 17, 18

e Mean computed from items 5, 6, 8, 11, 15, 16, 19

* Significance = *P* < 0.05;

** *P* < 0.01;

*** *P*<.001

On our weighted subdomain, the PBPT group showed a mean score of 1.84; the WBI group showed a mean score of 2.55 (*P* < 0.001), demonstrating patient‐relevant and ‐reported clinically significant differences between groups.

Significant differences are noted in Table 3. The PBPT group trended toward more responses indicating better outcomes, notably in “breast size disparity,” with mean scores of 1.74 and 2.34 for the PBPT and WBI groups, respectively (*P* < 0.001); “breast shape,” with mean scores of 1.60 and 2.25 for the PBPT and WBI groups, respectively (*P* < 0.001); “fit of clothing,” with mean scores of 1.06 and 1.464 for PBPT and WBI, respectively (*P* < 0.001); breast sensitivity, with mean scores of 1.44 and 1.95 for PBPT and WBI, respectively (*P* < 0.001); and breast texture, with mean scores of 1.44 and 1.91 for the PBPT and WBI groups, respectively (*P* < 0.001).

**Table 3:** BCTOS. Means of Breast Symptoms/Disparity affecting QoL reported by patients for questions displaying significant statistical differences between groups

Symptom/Disparity	PBPT (n = 72) Mean	WBI (n = 57) Mean	*P‐*value
Breast size^2,^ a	1.74	2.34	<0.001***
Breast texture^2^	1.44	1.91	<0.001***
Arm heaviness^4^	1.14	1.18	0.606
Nipple appearance^2^	1.41	1.70	0.051
Shoulder movement^1^	1.11	1.24	0.178
Arm movement^1^	1.10	1.21	0.161
Breast pain^3^	1.30	1.67	0.003**
Ability to lift objects^1^	1.14	1.30	0.119
Shirt sleeve fit^4^	1.04	1.20	0.052
Breast tenderness^3^	1.49	1.72	0.084
Shoulder stiffness^1^	1.08	1.13	0.441
Breast shape^2^	1.60	2.25	<0.001***
Breast elevation^2^	1.46	1.89	0.018*
Scar tissue^2,^ a	1.70	2.09	0.011**
Shoulder pain^1^	1.17	1.23	0.480
Arm pain^1^	1.11	1.19	0.252
Arm swelling^4^	1.07	1.04	0.396
Breast swelling^4^	1.04	1.07	0.618
Arm stiffness^1^	1.07	1.05	0.753
Fit of bra^2^	1.30	1.74	0.002**
Breast sensitivity^3,^ a	1.44	1.95	<0.001***
Fit of clothing^2^	1.06	1.46	<0.001***

Respondents graded the treated breast compared to untreated breast according to the following options: 1 = None; 2 = Slight; 3 = Moderate; 4 = Large (Major).

Question Categories (superscript numbers): ^1^Functional status; ^2^Cosmetic status; ^3^Breast Specific Pain; ^4^Edema.

Of those responding, women rated the most significant three questions

* Significance = *P* < 0.05;

** *P* < 0.01;

*** *P*<.001

### 3.4 Brief fatigue inventory

**Figure 3:**
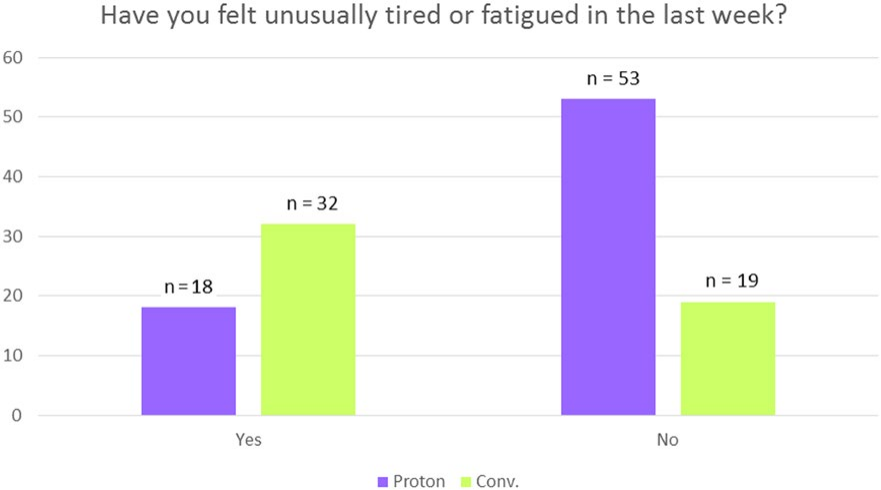
Frequencies of fatigue levels as reported by patients on the question “Have you felt unusually tired or fatigued in the last week?”

The average total BFI score (excluding question 1) was found to be 15.30 for the PBPT group and 27.25 for the WBI group, with standard deviations of 17.11 and 22.26, respectively, indicating less fatigue reported by the PBPT group (*P* < 0.002). The weighted Brief Fatigue Inventory was not significant and showed group mean scores of 3.12 and 3.90 for the PBPT and WBI groups, respectively, with standard deviation of 3.19 and 2.51, respectively (*P* < 0.531). Differences were significant (*P* < 0.001) in response to the question relating to how fatigue was perceived to affect daily lives. The score distribution for the first question of the Brief Fatigue Inventory is displayed in Figure 3. The individual questions covering fatigue, including intensity and frequency, are addressed in Table 4.

**Table 4:** Brief fatigue inventory

Question	PBPT (n = 72) Mean	WBI (n = 57) Mean	*P‐*value
Fatigue now	2.240	3.77	0.002**
Usual fatigue level in past 24 h	2.41	3.37	0.024*
Worst fatigue level in past 24 h	3.01	4.21	0.025*
Fatigue interference in activity, past 24 h			
General^a^	1.67	2.75	0.017*
Mood	1.14	2.77	<0.001***
Walking ability	1.78	2.41	0.192
Normal work (including chores)^a^	1.88	2.98	0.024*
Relations, other people	0.83	2.15	<0.001***
Enjoyment of life^a^	1.26	2.80	<0.001***

Means of fatigue symptoms/disparity affecting QoL. The table lists questions for which significant statistical differences were observed between groups. Respondents graded questions on a scale of 0 = No Fatigue to 10 = As bad as you can imagine.

a Of those responding, women rated the most significant three questions.

* Significance = *P* < 0.05;

** *P* < 0.01;

*** *P*<.001

### 3.5 Medical outcomes study short form survey

Of the 20 questions, significant differences were seen in six. General health questions, “would you say your health is …” displayed significance (*P* < 0.01) on a scale in which 1 = “Excellent” and 5 = “Poor”). On the subsection relating to emotional and social functioning, questions displaying significance in mean differences (*P* < 0.01) for PBPT and WBI groups were as follows: feeling “downhearted and blue”; feeling “like a happy person”.

On the subsection relating to perceptions of health, two questions displaying significance (*P* < 0.05; *P* < 0.01) and one highly significant (*P* < 0.001), respectively, were as follows: “I am somewhat ill”; “I am as healthy as anybody I know”; and “I have been feeling bad lately”.

Responses to other questions^20^ did not yield significant differences.

### 3.6 Body image scale

Overall, the PBPT group showed significantly better total scores for the Body Image Scale. The PBTI group showed an overall score of 12.04 and the WBI, 13.91, with standard deviations of 3.75 and 5.25, respectively (*P* < 0.03). Mean values from the PBPT group were significantly different on six of the ten questions asked (Table 5).

**Table 5:** Body image scale

Question	PBPT (total = 72) Mean	WBI (total = 57) Mean	*P‐*value
Feeling self‐conscious about appearance	1.38	1.77	0.004**
Feeling less physically attractive as a result of disease/treatment	1.25	1.49	0.060
Dissatisfied with appearance when dressed	1.19	1.49	0.005**
Feeling less feminine	1.13	1.26	0.136
Difficulty looking at self, naked	1.31	1.43	0.293
Feeling less sexually attractive	1.29	1.43	0.297
Avoiding people due to appearance	1.04	1.11	0.183
Feels body is “less whole”	1.08	1.25	0.047*
Dissatisfaction with body	1.26	1.56	0.007**
Dissatisfaction with scar appearance	1.21	1.46	0.048*

Means on questions related to body image, as reported by patients in each group. Respondents were asked to grade questions on a scale of 1 = Not at all; 2 = A little; 3 = Quite a bit; 4 = Very much.

* Significance = *P* < 0.05;

** *P* < 0.01;

*** *P*<.001

### 3.7 Relationships among domains

Table 6 presents correlation coefficients resulting from analyses of relationships among the various domains measured on validated scales. For all respondents, statistically significant correlations were found between several of the scales, indicating interrelationships among factors comprising patients’ perceptions of QoL. However, no correlations were seen between time since treatment (not shown in Table 6) or age and any score distributions on any of the scales used. A correlation was found between weight gain and cosmesis, cosmetic BCTOS, and Body Image Scale scores, but no significant difference was found between PBPT and WBI groups with respect to weight gain.

**Table 6:** Correlation coefficients resulting from analyses of relationships among domains measured via validated scales. The scales are identified in the left column and abbreviated in the top row**

	Age	WTCH	HCOS	WB	FUNC	COSB	BSP	EDB	TBFI	WBFI	BIS
Age	1	NA	0.017	0.027	0.065	0.033	−0.101	0.002	0.166	0.309	−0.027
Weight change (WTCH)	NA	1	0.248*	0.149	0.152	0.265*	0.400*	0.265*	0.119	−0.104	0.277*
Harvard Cosmesis Scale (HCOS)	0.017	0.248*	1	0.609*	0.016	0.490*	0.174	0.005	0.287*	0.117	0.184†
Weighted BCTOS (WB)	0.027	0.149	0.609*	1	468*	0.739*	0.513*	0.310†	0.284†	0.393	0.321†
Functional BCTOS (FUNC)	0.065	0.152	0.016	468*	1	0.840*	807*	0.915*	0.586*	0.312	0.819*
Cosmetic BCTOS (COSB)	0.033	0.265*	0.490*	0.739*	0.840*	1	0.842*	0.854*	0.668*	0.008	0.973*
Breast Specific Pain BCTOS (BSP)	−0.101	0.400*	0.174	0.513*	0.807*	0.842*	1	0.810*	0.580*	0.075	0.800*
Edema BCTOS (EDB)	0.002	0.265*	0.005	0.310†	0.915*	0.854*	0.810*	1	0.587*	−0.022	0.832*
Total Brief Fatigue Inventory (TBFI)	0.166	0.119	0.287*	0.284†	0.586*	0.668*	0.580*	0.587*	1	0.562*	0.629*
Weighted Brief Fatigue Inventory (WBFI)	0.309	−0.104	0.117	0.393	0.312	0.008	0.075	−0.022	0.562*	1	0.085
Body Image Scale (BIS)	−0.027	0.277*	0.184†	0.321†	0.819*	0.973*	0.800*	0.832*	0.629*	0.085	1

** Significant correlations are indicated by symbols.

* *P* < 0.001;

^†^ *P* < 0.05

### 3.8 General perspective

Several responses on this instrument indicated significant differences in the perceptions of PBPT and WBI patients (Table 7). The former group indicated greater happiness with their choice of treatment (mean 4.92 vs mean 4.20, *P* < 0.001); less perceived difference in how their skin felt since treatment (mean 1.22 vs, mean 1.95, *P* < 0.001); a lesser change in attitude about sex (mean 1.41 vs mean 1.94, *P* = 0.012); less of a change in perception of self and body (mean 1.57 vs mean 2.16, *P* = 0.008); and less worry about recurrent cancer (mean 2.31 vs mean 3.27, *P* < 0.001).

**Table 7:** Means of responses to “General Perspective” questions

Question	PBPT (n = 72) Mean	WBI (n = 57) Mean	*P‐*value
Happy with treatment choice	4.92	4.20	<0.001***
Skin quality during treatment	1.50	2.82	<0.001***
Skin “felt different” since treatment	1.22	1.95	<0.001***
Changed attitude about sex	1.41	1.94	0.012*
Breast cancer changed views of “myself and body”	1.57	2.16	0.008**
Worry about “disease coming back”	2.31	3.27	<0.001***
Changed how I live daily life	2.00	2.30	0.197
Role of Spirituality/Religion	4.35	4.00	0.116
Upper arm/mobility issues	1.19	1.30	0.348

Respondents were asked to rate each question on a scale of 1 = Not at all to 5 = Very much.

* Significance = *P* < 0.05;

** *P* < 0.01;

*** *P* < 0.001

## 4 DISCUSSION

Our data are the first to analyze long‐term radiation‐related QoL across physical and emotional domains in women with EBC treated with PBPT or WBI as part of BCT. Some data confirm a study by our team,^1^ in which cosmetic satisfaction results of 90% were maintained through follow‐up.

According to our data, PBPT patients reported less pain, less fatigue, fewer restrictions in daily activities, and better cosmetic results. Further, superior perceived QoL outcomes on the various domains surveyed tended to correlate strongly with other corroborating domains, suggesting that PBPT led to a cascading series of favorable outcomes.

### 4.1 Consideration of factors

In contrast to the RAPID trial comparing WBI with 3DCRT APBI, demonstrating worse cosmesis and normal tissue toxicity for the 3DCRT APBI group,^23^ better cosmesis outcomes were found in the PBPT group in our study. These results are likely due to the volume of breast irradiated in each modality, a supposition that accords with the study of Jagsi et al,^24^ in which volume of breast irradiated via a partial breast photon technique correlated with adverse cosmesis. Moreover, there were significant differences not only in cosmesis, but also on functional BCTOS and fatigue measures, again plausibly due to a smaller volume irradiated via PBPT. The Body Image Scale results for PBPT patients and the positive correlations between cosmesis scores and Body Image Scale scores confirms and elaborates on differences found for cosmesis measures between groups; physical differences relate to differences in emotional, social, and sexual QoL.

Additionally, results on the Brief Fatigue Inventory and MOS help to support the hypothesis that not only does volume irradiated have functional effects, but that these differences adversely affect patients’ activities of daily life, thus affecting their social and emotional health. In contrast to studies which suggest that differences found between PBPT and WBI are most pronounced during treatment period and immediate follow‐up, but converge to insignificance in the long term,^11^, ^16^ our results suggest that the significant differences remaining between groups are entirely due to the difference in treatment modality.

A key strength of this study is the long‐term nature of the follow‐up for PBPT patients, which establishes a more comprehensive understanding of the QoL trend profile for PBPT. A further strength lies in the comprehensive evaluation, yielding a holistic picture of QoL that provides not only a more robust assessment of patient outcomes but also demonstrates that none of these measures can be evaluated in a vacuum without considering the interconnected factors affecting QoL.

### 4.2 Potential confounding factors

Despite these promising findings, this is a correlational study. Accordingly, alternative explanations and possible confounding factors must be considered. While it is proposed that differences in cosmesis, function, pain, and fatigue yielded differences in body image perception, perception of fatigue interference with daily life, and emotional and social health, the causal mechanism could have acted the opposite direction, so that women having a generally negative emotional outlook would perceive fatigue, cosmesis, pain, and function to be worse. On the other hand, all but two of the PBPT participants participated in a clinical trial; this fact might have led them to perceive PBPT as more effective, thus altering their perceptions of their treatment outcomes in all measures, because some, or many, may have surmised that they were receiving the most advanced treatment for their disease.

**Figure 4:**
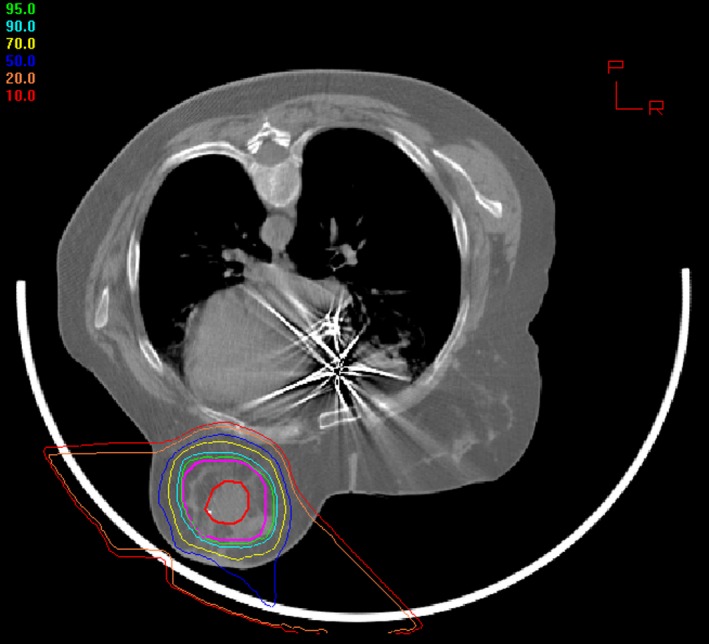
Dose distribution in patient treated with PBPT. The target volume is circled in red. The patient is treated in the prone position, with the untreated breast compressed away from the beam. The heart, lungs, and untreated breast receive no radiation

Additionally, lumpectomies were performed by a variety of surgeons, at Loma Linda and elsewhere, so patients’ perceptions of QoL may have been affected by the quality of their initial surgical procedures. Table 1 attempts to cover some aspects of the surgical procedures performed. Lastly, the longer distances traveled by PBPT patients (Table 1) may imply that many of these patients might have sought out this treatment and thus had a mindset that affected differences in long‐term evaluations of outcomes across domains. Further, several women reported anecdotally that they selected PBPT because they learned on their own that the modality can preserve critical structures (Figure 4). Such women could have a predisposition to regard PBPT positively. A parallel may exist with findings of Talcott et al^25^: those seeking PBPT were a motivated group who wanted their treatment to succeed.

A future study may be required to investigate the effects of patients’ initial opinions about different treatment options, their subsequent outcome evaluations, and the effects of emotional and social health on other QoL measures.

Our population, comprising mostly Caucasian, well‐educated (ie, college education or higher), married, urban women, may not be generalizable to patients of other racial/ethnic, educational, or demographic backgrounds. Even so, population characteristics were consistent between the two groups. Our sample size is small and was derived from patients treated at one institution, thus suggesting the potential need to verify findings in a larger, multi‐institutional study.

### 4.3 Consideration of interrelating factors

The normal tissue‐sparing aspect of PBPT, whether patients had foreknowledge of it or not, may affect patients’ QoL perceptions. For example, maintaining function, eliminating pain, and sparing vital organs may lead to less fatigue later, resulting in greater everyday functioning and participation in meaningful activities, further affecting social and emotional outlook. Using PBPT to spare part of the treated breast, as well as the opposite breast, will, compared to WBI, result in differences in appearance, texture, feel, and comfort. These differences may result in different evaluations of cosmesis, leading to perceived differences in body image and affecting, perhaps, social, and emotional functioning.

Patients’ QoL perceptions may also be related to their treatment option. PBPT, for example, may concern some about risk of recurrence because only part of the breast is irradiated^12^; others may favor PBPT because the shorter overall treatment time enables them to resume daily living sooner. Such attitudinal differences may affect confidence in one's treatment, in turn influencing social and emotional outcomes. Notably, Albuquerque et al,^9^ investigating the impact of whole breast vs accelerated partial breast irradiation on fatigue, stress, and overall QoL, found that patients’ concept of well‐being may be influenced by distance traveled to and from treatment, and by decreased treatment length; they, and others,^12^ also describe associated implications for cost, time off work, transportation, and providing for child/family care.^12^ Belkacemi et al^10^ describe shortened treatment duration as contributing to fewer distressing factors for elderly patients, thus affecting compliance. Our data show similar trends.

Cosmesis essentially means comparing the treated breast to the untreated breast. We used two patient‐reported outcome (PRO) measures to report on this: cosmesis and the BCTOS. Studies on the development and results of a radiation‐specific breast symptom measure^5^, ^26^ report that objective and subjective indicators of cosmetic and functional status correlate many years after treatment.

Patient‐reported outcomes from PBPT patients revealed significant differences in perceived QoL, compared to X‐ray patients. Broadly, the differences are in perceived cosmesis (body image, breast appearance), and comfort/functionality (fit of clothes, pain, feeling fatigued, and breast tenderness/hardness). On our weighted subdomain, differences in BCTOS scores reflect patients’ subjective experiences and the differences seen between treatment groups on factors most important to patients. Interaction analyses showed degrees of covariance such that higher (worse) scores in BCTOS subdomains were correlated with lower (worse) scores on Body Image and Fatigue inventories (Table 6).

We also found correlations between comfort and fatigue. PBPT‐treated women reported significantly more‐favorable scores on these factors. Fatigue may arise from restricted functionality or pain; pain and functionality domains were correlated with fatigue scores.

Our respondents are over five years post‐treatment. All effects reported are long‐term secondary effects. Our data show that some such effects either persist or occur five to ten years later. Differences by time since diagnosis during this window, however, are insignificant correlates for any symptoms; therefore, after five years (at the latest) it seems likely that these symptoms stabilize. Nonetheless, long‐term differences between groups remain and most likely owe to differences in treatment.

### 4.4 Possible implications for patients considering treatment

Ideally, discussions between providers and patients include how other women's QoL, and potential short‐ and long‐term treatment effects, relate to different BCT approaches. Although women adjust to breast cancer and its treatment, many experience long‐term consequences: later effects may emerge, some lingering; these, coupled with aging and/or use of endocrine therapy, can negatively affect QoL.^27^ Accordingly, as women experience long‐term survival, clinicians and patients share the goal to maintain or improve QoL. Our study has shown the interrelationship of factors respondents perceived as important in QoL. Collectively, we believe these results reinforce the initial construct of our investigations (Figure 1).

Patient‐reported outcomes employed in measurement‐focused comparative research provide essential data for clinicians, payers, and health policymakers. Information obtained therefrom benefits patients and practitioners by assisting in clinical decisions, assessing patients’ ongoing needs, and understanding patients’ preferences.^28^, ^29^, ^30^

Our results indicate a QoL advantage for PBPT in EBC. Proton radiation therapy, long used clinically, is expanding: 27 proton treatment centers now operate in the United States and 10 more are under construction.^31^ Long‐term studies^18^ confirm that protons conform the dose to the tumor and avoid healthy surrounding structures,^32^ leading to fewer side effects and, accordingly, improved QoL. At our institution, we now offer PBPT as standard‐of‐care treatment for EBC patients.

## 5 CONCLUSION

According to patient reports in our survey, QoL in PBPT‐treated women is, at 5‐10 years post‐treatment, significantly better than those treated with WBI for all domains analyzed. This study is unique in its long‐term scope and the comprehensive, holistic, and patient‐focused model of QoL, which is crucial to understand the complex reality of the healthcare process on the lives of patients treated. PBPT patients reported less pain, less fatigue, fewer restrictions in daily activities, and better cosmetic results over several corroborating domains. Results confirm that PBPT is not only an effective BCT treatment option for early‐stage disease, but that it also presents significantly improved overall outcomes many years out from treatment, across many domains. These quality‐of‐life differences are increasingly important as women are surviving many more years post‐treatment; thus, this study bears particular relevance to this shift of focus. PBPT is not only an effective treatment for managing disease, but is an advantageous option for minimizing the impact of breast cancer and its treatment on daily life, not merely in the short term, but even for several years after treatment.

## CONFLICT OF INTEREST

Actual or potential conflict of interest do not exist. Patient photos have not been used. Copyrighted material has not been used.
